# Case of a novel self‐assembling peptide hemostatic gel as a therapeutic tool for endoscopic ultrasound‐guided tissue acquisition‐related bleeding

**DOI:** 10.1111/den.14541

**Published:** 2023-04-04

**Authors:** Masanari Sekine, Keita Matsumoto, Hirosato Mashima

**Affiliations:** ^1^ Department of Gastroenterology, Saitama Medical Center Jichi Medical University Saitama Japan

## Abstract

Watch a video of this article.

## Brief Explanation

Endoscopic ultrasound‐guided tissue acquisition (EUS‐TA) is a very important and useful method in the clinical setting. Bleeding is one of a few adverse events for EUS‐TA.^1^ Recently, a novel self‐assembling peptide hemostatic gel was reported to be safe and effective against bleeding.^2^, ^3^, ^4^, ^5^ We herein describe a case of endoscopic hemostasis for post‐EUS‐TA bleeding using a novel hemostatic gel.

**Figure 1:**
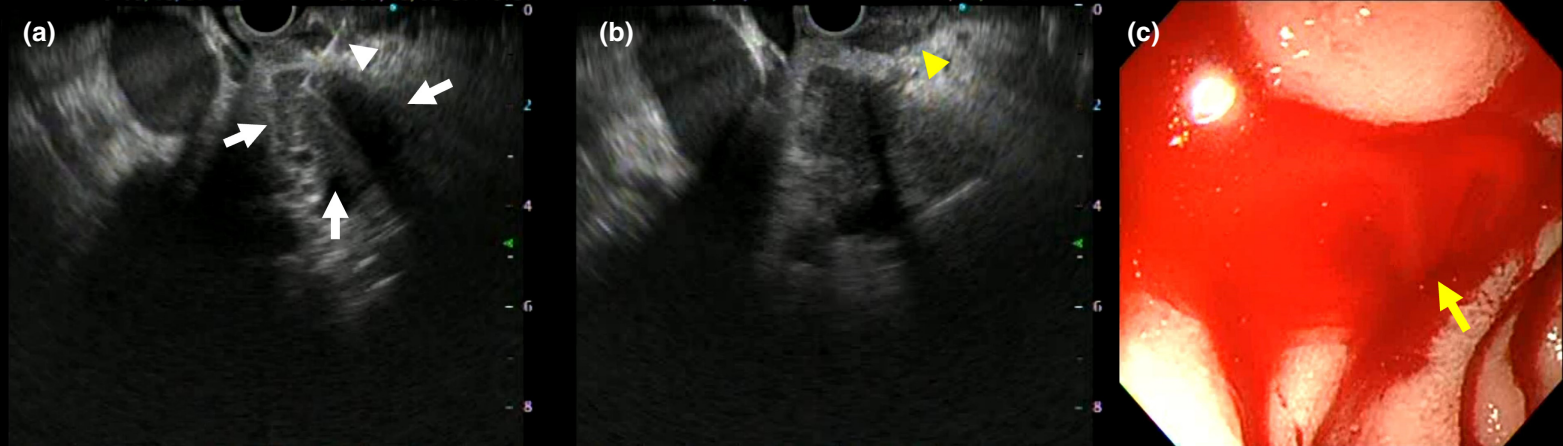
(a) Endoscopic ultrasound‐guided tissue acquisition was performed for a pancreatic tumor (white arrows) from the duodenal bulb using a 25G Franseen needle (white arrowhead). (b) There was no intraperitoneal or intramural bleeding (yellow arrowhead). (c) Spurting hemorrhage occurred from the puncture site (yellow arrow).

**Figure 2:**
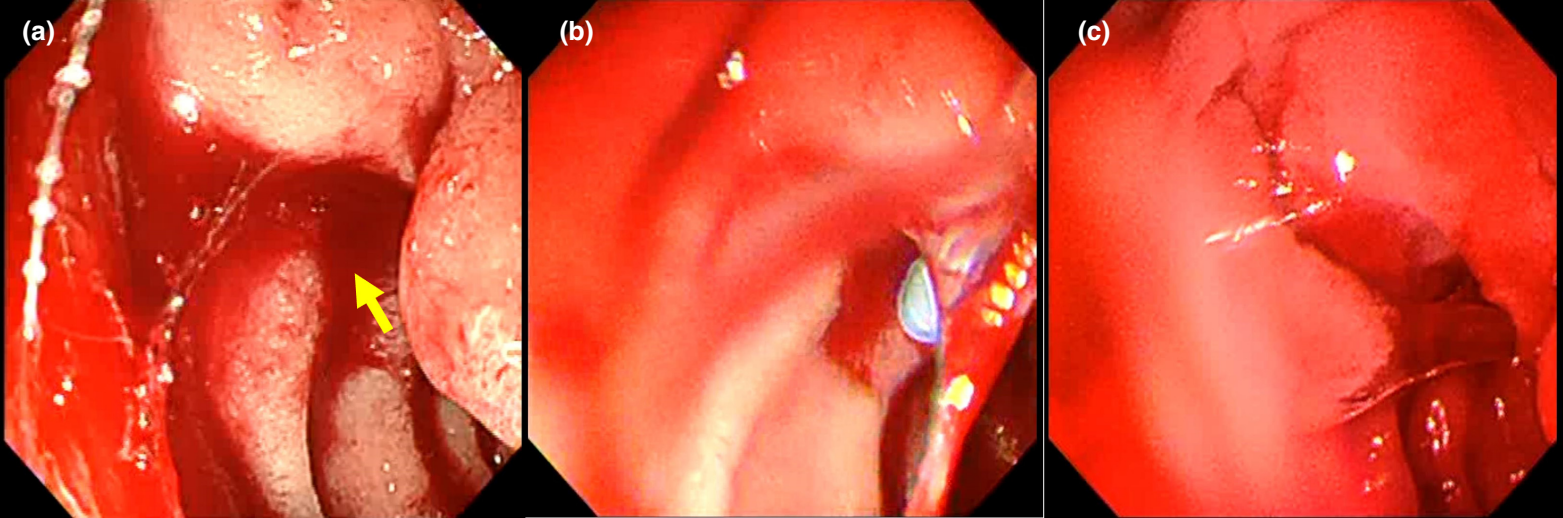
(a) After pressing the bleeding point with the endoscopic ultrasound scope, the bleeding was weakened to oozing (yellow arrow); (b) a novel self‐assembling peptide hemostatic gel (PuraStat; 3‐D Matrix, Tokyo, Japan) was subsequently applied. (c) Hemostasis was successfully achieved.

The patient was a 66‐year‐old man. The biliary and pancreatic stents were inserted for obstructive jaundice at another hospital. Bile duct biopsies for the stenosis were performed at that time, not leading to a histological diagnosis of malignancy. Therefore, the patient was transferred to our hospital. The patient continued heparin after switching from warfarin. We visualized a hypoechoic mass (20 mm in diameter) in the pancreatic head in EUS and suspected pancreatic cancer. We performed EUS‐TA from the duodenal bulb using a 25G Franseen needle, because the intramural vessel and the gastroduodenal artery intervened on the puncture route (Video S1). Spurting hemorrhage was observed in the duodenal cavity after the puncture (Fig. 1). At first, we confirmed that there were no intraperitoneal and intramural bleeding by EUS. We then pressed the bleeding point with the EUS scope. After confirming that the bleeding was weakened to oozing, we sprayed 3 mL of a novel self‐assembling peptide hemostatic gel (PuraStat; 3‐D Matrix, Tokyo, Japan) and confirmed the hemostasis (Fig. 2).

For endoscopically visible bleeding without intramucosal bleeding, pressing the bleeding point with the EUS scope is useful. If the bleeding nevertheless does not subside, hemoclips may be needed. In such a situation, a novel self‐assembling peptide hemostatic gel can be an effective and safe option for hemostasis.

Authors declare no conflict of interest for this article.

## INFORMED CONSENT

The subject of the case report provided informed consent to publish the included information.

## Supporting information


**Video S1** Novel hemostatic gel for post‐endoscopic ultrasound‐guided tissue acquisition bleeding.
