# Supplementary material for: Case of a novel self‐assembling peptide hemostatic gel as a therapeutic tool for endoscopic ultrasound‐guided tissue acquisition‐related bleeding
Source: Dig Endosc. 2023 Apr 4;35(4):e70–1. doi: 10.1111/den.14541 (PMC12136271; doi:10.1111/den.14541)
Supplement: Supplementary file 1 — Video S1 Novel hemostatic gel for post‐endoscopic ultrasound‐guided tissue acquisition bleeding. [file DEN-35-e70-s001.docx]

1，

We visualized a hypoechoic mass (20mm) in the pancreatic head by EUS and suspected pancreatic cancer.

An endoscopic biliary stent (EBS) and an endoscopic pancreatic stent (EPS) had been inserted in a mildly dilated bile duct and pancreatic duct.

2,

We performed EUS-TA from the duodenal bulb using a 25G Franseen needle.

3,

After the puncture, we observed spurting-type bleeding in the duodenal cavity.

At first, we confirmed that there was no intraperitoneal and intramural bleeding by EUS.

After that, we pressed the bleeding point with the EUS scope.

4,

We observed oozing-type bleeding.

In addition, we pressed the bleeding point more on EUS scope.

5,

We confirmed the bleeding had weakened.

We applied 3 ml of hemostatic gel to the source of the bleeding using an endoscopic catheter. Hemostasis was achieved.
